# Impact of Ophthalmic Viscosurgical Devices in Cataract Surgery

**DOI:** 10.1155/2020/7801093

**Published:** 2020-10-20

**Authors:** Monali S. Malvankar-Mehta, Angel Fu, Yasoda Subramanian, Cindy Hutnik

**Affiliations:** ^1^Department of Ophthalmology, Schulich School of Medicine and Dentistry, The University of Western Ontario, London, ON, Canada; ^2^Department of Epidemiology and Biostatistics, Schulich School of Medicine and Dentistry, The University of Western Ontario, London, ON, Canada; ^3^Faculty of Medicine, University of Ottawa, Ottawa, ON, Canada; ^4^Royal College of Surgeons in Ireland, Dublin, Ireland

## Abstract

**Background:**

Ophthalmic viscoelastic devices (OVDs) used during small-incision cataract surgery have numerous advantages. However, OVDs have longer retention time in an eye after surgery resulting in intraocular pressure (IOP) spikes. The purpose of this study is to analyze and quantify the effect of various OVDs on both IOP and best corrected visual acuity (BCVA) by systematically reviewing the literature and performing meta-analysis.

**Methods:**

Numerous databases from January 1, 1985, to present were systematically searched. Thirty-six (3893 subjects) of 3313 studies identified were included for analysis. Standardized mean difference (SMD) was computed, and meta-analysis was performed.

**Results:**

A total of 3313 records were retrieved including 1114 from database search and 2199 from grey literature search. Significant increase in postoperative IOP in 1-day follow-up with Healon (SMD = 0.37, CI: [0.07, 0.67]), Viscoat (SMD = 0.29, CI: [0.13, 0.45]), Provisc (SMD = 0.46, CI: [0.17, 0.76]), and Soft Shell (SMD = 0.58, CI: [0.30, 0.86]) was computed. On the other hand, results implied a nonsignificant increase in postoperative IOP with Healon GV (SMD = 0.07, CI: [−0.28, 0.41]), Healon5 (SMD = 0.15, CI: [−0.33, 0.64]), 2% HPMC (SMD = 0.32, CI: [−0.0, 0.64]), and OcuCoat (SMD = 0.26, CI: [−0.37, 0.9]). Additionally, a nonsignificant reduction in postoperative IOP was inferred with Viscoat + Provisc (SMD = −0.28, CI: [−2.23, 1.68]).

**Conclusion:**

Improvement in IOP was shown with Viscoat + Provisc. Additionally, IOP nonsignificant upsurge was observed with Healon GV, Healon5, 2% HPMC, and OcuCoat compared to significant upsurge with Healon, Viscoat, and Soft Shell.

## 1. Introduction

A small-incision cataract surgery is the preferred method of cataract surgery by most surgeons. Ophthalmic viscoelastic devices (OVDs) have numerous advantages during small-incision cataract surgery. OVDs protect corneal endothelium against fluid turbulence, oxygen free radicals released during ultrasound [1], contact with surgical instruments, air bubbles, and lens fragmentation [2]; facilitate surgical procedure; reduce the risk of collateral damage to delicate intraocular tissues [3], maintain the anterior chamber space and stability to avoid capsular rupture [3]; and provide clarity to avoid complications. These properties may vary based on physical, chemical, and rheological characteristics of OVDs [4]. An ideal OVD [5]—which does not exist yet—would be easy to inject into the eye, would maintain anterior chamber [6], would not impair vision by trapping air bubbles, would not increase intraocular pressure (IOP), and would be easy to remove from the eye after the surgery.

However, a major disadvantage of OVDs is longer retention time in an eye after cataract surgery resulting in IOP spikes [7–10]. OVDs remain in the eye resulting in mechanical obstruction of the trabecular meshwork, impeding outflow and causing IOP spikes within 24 hours after surgery which have become a concern [10], specifically for glaucoma patients. In glaucoma patients, IOP spikes may cause significant damage to the optic disc [9].

In the literature, numerous prospective randomized control trials (RCTs) have been conducted to compare safety, efficacy, and performance of various OVDs used during routine small-incision cataract surgeries and intraocular lens (IOL) implantation. Most studies compared preoperative characteristics including best corrected visual acuity (BCVA), central corneal thickness (CCT), endothelial cell count (ECC), and IOP with postoperative characteristics in various OVDs at several time intervals. Few studies suggested that super viscous and cohesive OVDs take longer time to remove from a normal eye leading to IOP elevation for a greater time period [3, 4, 11, 12]. On the other hand, few studies state that longer aspiration time is required to remove dispersive OVDs [1] compared to cohesive OVDs [1, 10, 13].

In 2009, a single study [14] evaluated the protective effect of different viscoadaptive, super viscosity cohesive, viscous cohesive, medium viscosity dispersive, very low viscosity dispersive, and Soft Shell devices on ECC during cataract surgery by conducting a meta-analysis. Our research expands by evaluating the effect of various OVDs on IOP and BCVA during small-incision cataract surgery by performing a systematic review and meta-analysis. For systematic review, published as well as unpublished (grey) literature is systematically searched and data is synthesized from the included articles to compare various OVDs in order to investigate the answer to two key questions: (1) Which OVD causes the greatest risk of an IOP spike? (2) What is the specific postoperative time point at which an IOP spike is most likely to occur?

## 2. Methods

### 2.1. Search Strategy

In this work, we adhered to the Preferred Items for Systematic Reviews and Meta-Analyses (PRISMA) guidelines [15]. Bibliographic databases, including MEDLINE (OVID and PubMed), EMBASE (OVID), BIOSIS Previews (Thomson-Reuters), CINAHL (EBSCO), Health Economic Evaluations Database (HEED), ISI Web of Science (Thomson-Reuters), and the Cochrane Library (Wiley) till December 2018, were searched. Database specific subject headings and key words for “ophthalmic viscoelastic device” or “ophthalmic viscosurgical device,” and “increased IOP” or “endothelial cell loss” were employed in the search strategy. The searches were modified to accommodate syntax of each database (S2). OVID AutoAlerts were set up to send monthly updates with any new literature.

For grey literature, various conference abstracts including the Canadian Ophthalmology Society (COS) meeting, American Academy of Ophthalmology (AAO) annual meeting, European Society of Ophthalmology (SOE), and the Association for Research in Vision and Ophthalmology (ARVO) annual meeting were searched. Additionally, ProQuest Dissertations and Theses database and the Canadian Health Research Collection (Ebrary) were searched.

### 2.2. Selection Criteria

**Figure 1:**
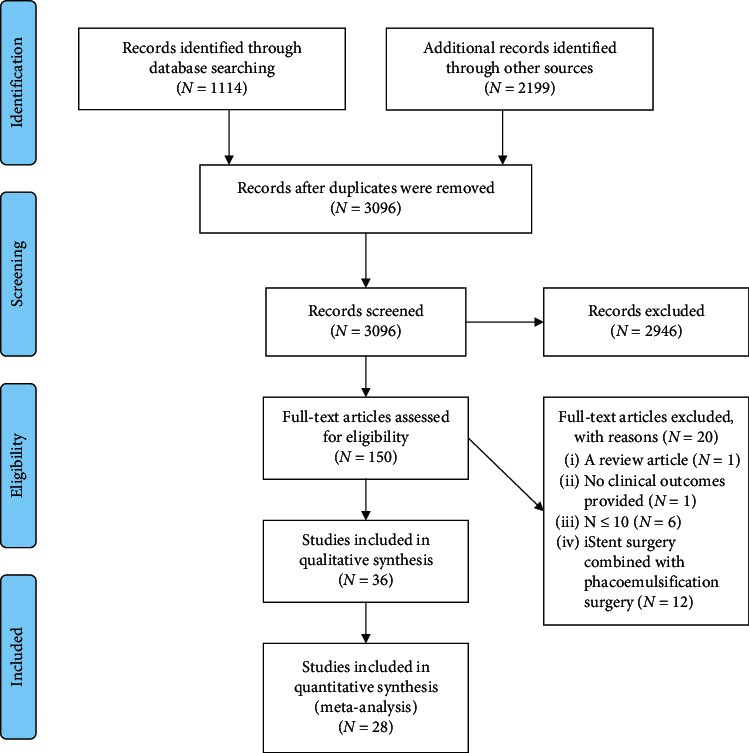
PRISMA 2009 flow diagram (from [15]; for more information, visit http://www.prisma-statement.org).

Randomized controlled trials, published in English language, discussing unilateral and bilateral cataract surgery on human subjects above the age of 19 and older were included. No restriction was placed based on study location.  Figure 1 summarizes the PRISMA flow diagram.

In total, 1114 records were retrieved from multiple databases including MEDLINE (213), EMBASE (639), ISI Web of Science (195) Cochrane Library (5), and CINAHL (62). An additional 2,199 records were identified through grey literature searches. EPPI-Reviewer 4 gateway (by EPPI-Centre, Social Science Research Unit, the Institute of Education, University of London, UK) was used to conduct the systematic review. All identified records were imported to EPPI-Reviewer 4 to remove duplicates. After removing duplicates (217 records), 3,096 records were included for the three-level screening process.

### 2.3. Screening

Tittle (Level 1) screening involved reviewing titles, while abstract (Level 2) screening involved reviewing abstracts, and full-text (Level 3) screening (S3) involved full-text reviews of included articles, independently by two reviewers (AF and YS). At each level, agreement and disagreement between the two reviewers were assessed by Cohen's kappa (*κ*) coefficient. Differences between the reviewers were discussed and resolved by consensus. In cases where consensus was not achieved, a third reviewer (MM) intervened to provide a decision.

### 2.4. Data Extraction

Data was extracted from the 36 eligible articles using a data extraction form. Data included study design, location, total patients enrolled, total patients enrolled in and completed the study, number of females, patient demographic characteristics, follow-ups, and baseline and postoperative characteristics including IOP, BCVA, CCT, and ECC. For missing data, various pieces of available information (such as the range, *p* value, and confidence interval) were utilized and converted to the common effect measure—SD. Quality of each included article was checked using modified Downs and Black checklist [16].

### 2.5. Meta-Analysis

Meta-analysis was conducted using STATA v. 15.0. (STATA Corporation, College Station, TX). By statistically combining the pre- and postoperative IOP and BCVA from included studies, the power of the analysis significantly increased, resulting in a single summary effect estimate of IOP and BCVA.

Standardized mean difference (SMD) was computed as the effect size. The extracted mean and standard error of the IOP at baseline and end point were used to compute the mean IOP reduction (IOPR), percentage of IOP reduction (IOPR%), and standard deviation percentage of IOP reduction (SD_IOPR%_) [17].

SMD was calculated as the treatment effect since it is a mean difference standardized across all studies. Weights were assigned to each SMD according to the inverse of its variance, and then average was computed. SMD for each study was then aggregated using the fixed or random-effect model based on the presence of heterogeneity to estimate the summary effect.

To test heterogeneity, *I*^2^ statistics, *Z*-value, and *χ*^2^ statistics were computed. Additionally, a high *Z*-value, a low *p* value (<0.01), and a large *χ*^2^ value imply significant heterogeneity and, therefore, a random-effects model using DerSimonian and Laird methods was computed. Funnel plots were generated to check publication bias.

Subgroup analysis was conducted to ascertain the influence of OVD following the cataract surgery on postoperative IOP and BCVA. Causes of heterogeneity were also explored.

## 3. Results

### 3.1. Search Results

Total of 36 RCTs were eligible for meta-analysis (Table 1). From these 36, 28 RCTs had data on IOP (S4), 13 studies had data on central corneal thickness (CCT), 16 studies had data on endothelial cell count (ECC), 9 studies reported data on best corrected visual acuity (BCVA) (Table 1), 10 studies listed wash-out times for OVDs (Table 2), and 9 studies reported the adverse events that occurred postoperatively (Table 3). In the end, a total of 36 studies (3893 subjects) were included for qualitative synthesis and 28 studies (2613 subjects) for quantitative synthesis (Figure 1).

### 3.2. Publication Bias

**Figure 2:**
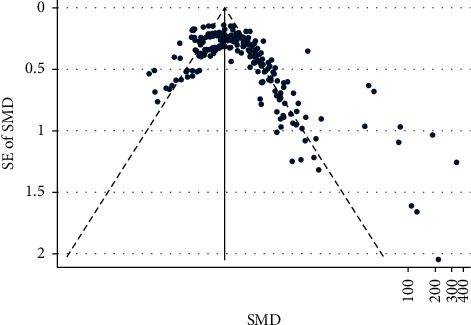
IOP funnel.

**Figure 3:**
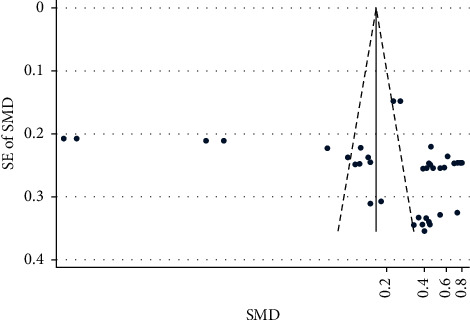
BCVA funnel.

Figures 2 and 3 show the funnel plots (S4) for studies reporting preoperative and postoperative IOP and BCVA, respectively, for various OVDs.  Figure 2 shows studies scattered from top to bottom right of the plot. Therefore, publication bias could not be concluded. Partially, the reason was difficulty in interpretation of funnel plot due to high heterogeneity and small effect sizes.

### 3.3. Main Outcomes

#### 3.3.1. Effect on Intraocular Pressure

**Figure 4:**
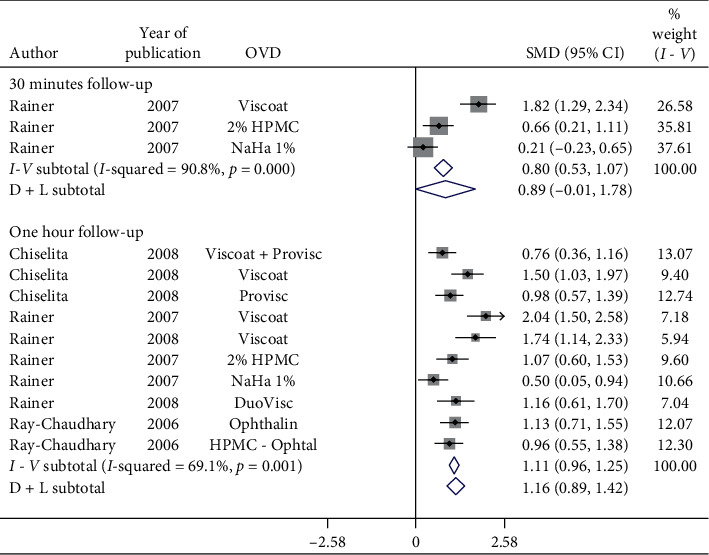
Forest plot for studies examining pre- and postoperative intraocular pressure (IOP) by 30-minute and one-hour follow-up.

Figure 4 shows a forest plot of SMD of pre- and postoperative IOP by follow-up. A single study evaluating various OVDs showed a nonsignificant increase in the postoperative IOP in 30-minute follow-up (SMD = 0.89, CI: [−0.01, 1.78]). However, further research is required to make robust inferences. For studies investigating the effect of OVDs in 1-hour follow-up, significant (*p*=0.001) heterogeneity between studies (*I*^2^ = 69.1%) was observed and, therefore, the random-effects model showed significant increase in IOP in 1-hour follow-up (SMD = 1.16, CI: [0.89, 1.42]).

**Figure 5:**
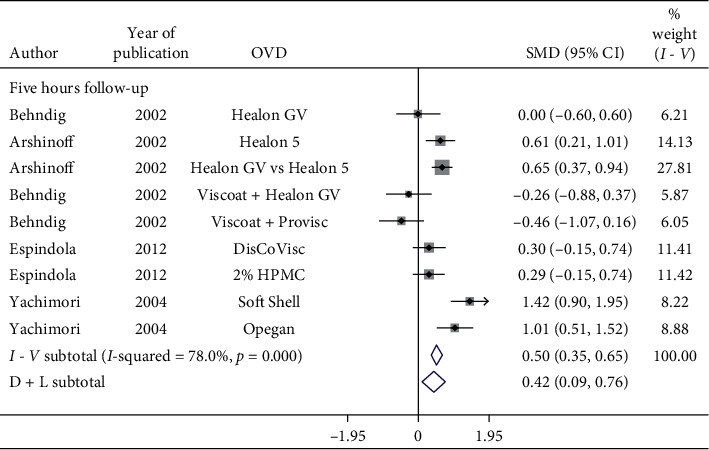
Forest plot for studies examining pre- and postoperative intraocular pressure (IOP) by five-hour follow-up.

Significant increase in postoperative IOP was observed in 2-hour, 3-hour, 4-hour, and 5-hour follow-up (SMD = 0.42, CI: [0.09, 0.76]) (Figure 5). Irrespective of the OVDs used, postoperative IOP in 4-hour follow-up does increase significantly. However, Viscoat + Healon GV and Viscoat + Provisc significantly reduce IOP in 5-hour follow-up (Figure 5). More studies evaluating Viscoat + Healon GV and Viscoat + Provisc are required.

**Figure 6:**
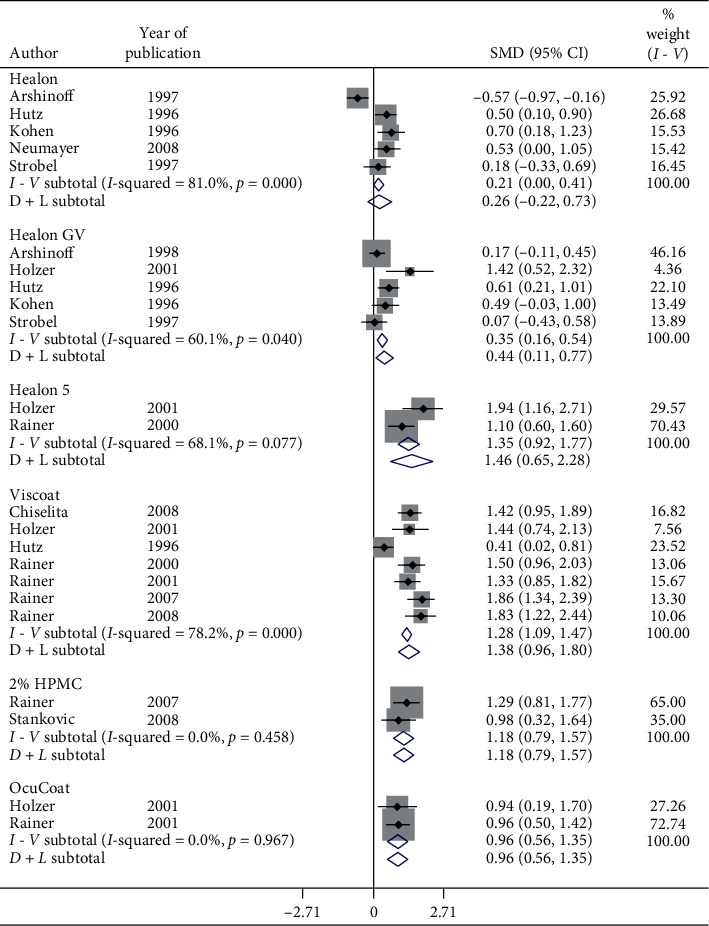
Forest plot for studies examining pre- and postoperative intraocular pressure (IOP) by six-hour follow-up and ophthalmic viscoelastic devices (OVDs).

**Figure 7:**
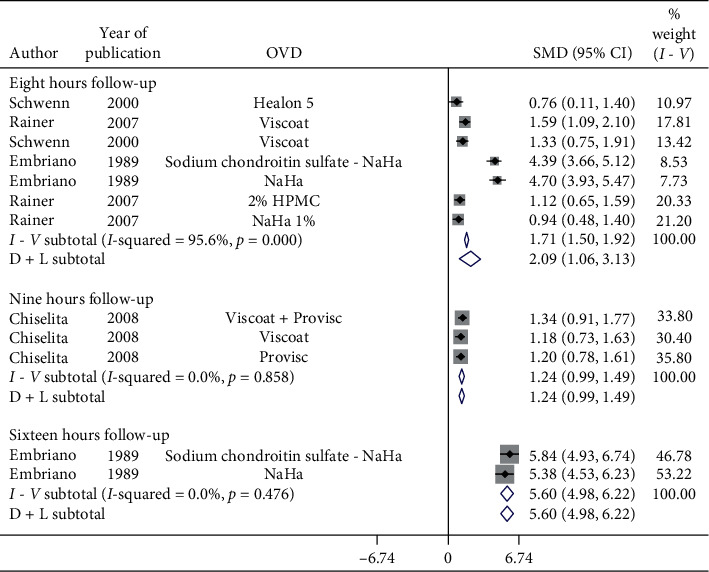
Forest plot for studies examining pre- and postoperative intraocular pressure (IOP) by eight-, nine-, and 16-hour follow-up.

Figure 6 shows a forest plot for subgroup analysis by OVDs for SMD of pre- and postoperative IOP in 6-hour follow-up in patients with cataract. Considerable heterogeneity between studies existed. Postoperative IOP significantly increased in 6-hour follow-up with Healon GV (SMD = 0.44, CI: [0.11, 0.77]), Healon5 (SMD = 1.46, CI: [0.65, 2.28]), Viscoat (SMD = 1.38, CI: [0.96, 1.8]), 2% HPMC (SMD = 1.18, CI: [0.79, 1.57]), and OcuCoat (SMD = 0.96, CI: [0.56, 1.35]) compared to a nonsignificant increase with Healon (SMD = 0.26, CI: [−0.22, 0.73]). Healon GV, Healon5, Viscoat, 2% HPMC, and OcuCoat may significantly increase IOP in 6-hour follow-up compared to a nonsignificant increase with Healon. Results showed significant increase (Figure 7) in IOP in 8-hour (SMD = 2.09, CI: [1.06, 3.13]), 9-hour (SMD = 1.24, CI: [0.99, 1.49]), and 16-hour follow-up (SMD = 5.6, CI: [4.98, 6.22]).

**Figure 8:**
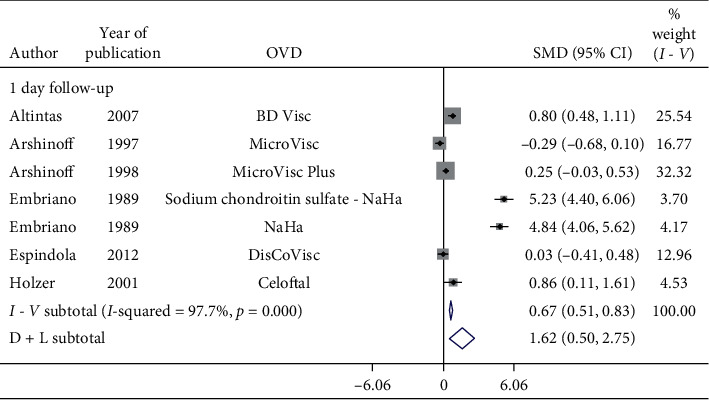
Forest plot for studies examining pre- and postoperative intraocular pressure (IOP) by 24-hour follow-up and ophthalmic viscoelastic devices (OVDs).

**Figure 9:**
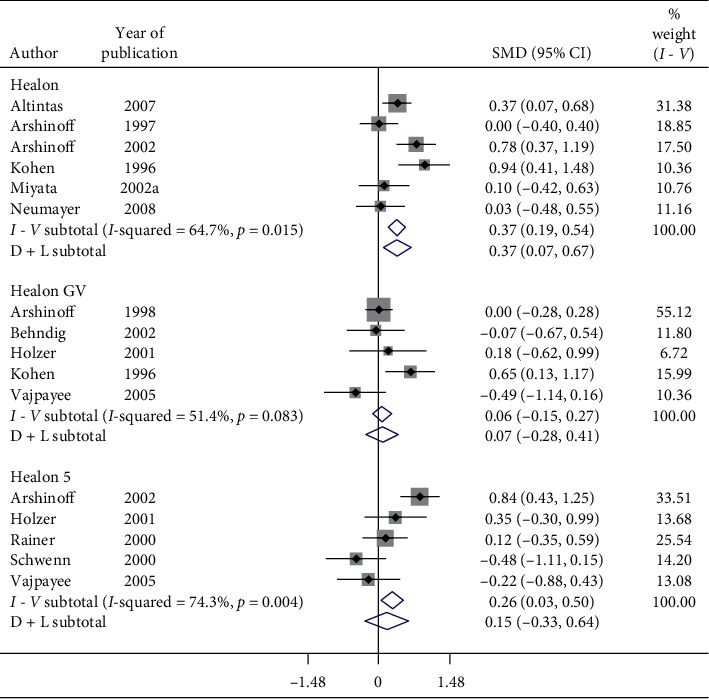
Forest plot for studies examining pre- and postoperative intraocular pressure (IOP) by 24-hour follow-up and ophthalmic viscoelastic devices (OVDs).

**Figure 10:**
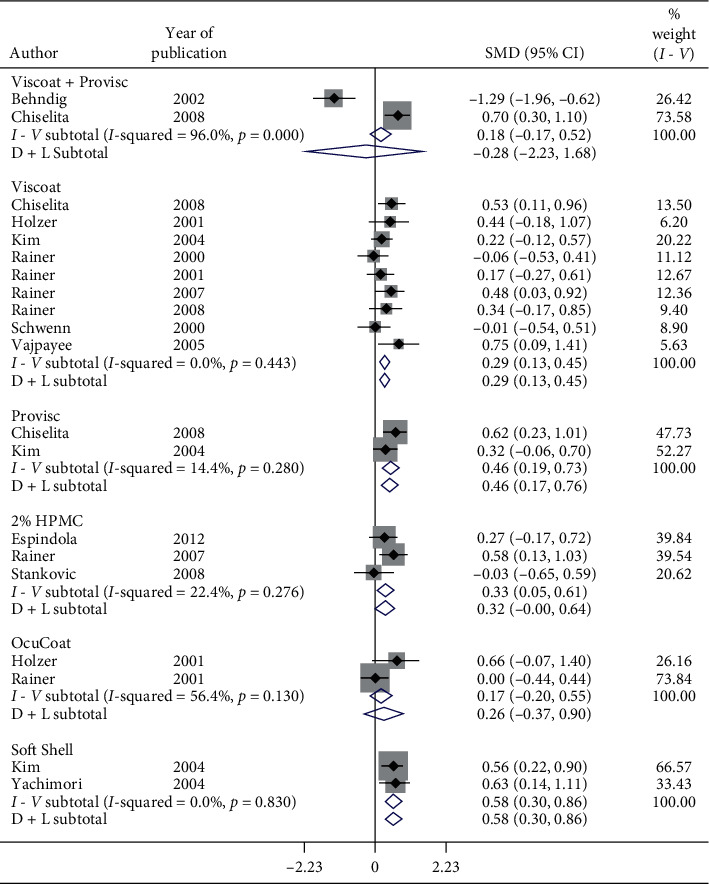
Forest plot for studies examining pre- and postoperative intraocular pressure (IOP) by 24-hour follow-up and ophthalmic viscoelastic devices (OVDs).

Subgroup analysis of postoperative IOP in 1-day follow-up by OVDs is shown in Figures 8–10. Considerable heterogeneity between studies existed. Significant increase in postoperative IOP (Figures 8 and 9) in 1-day follow-up with Healon (SMD = 0.37, CI: [0.07, 0.67]), Viscoat (SMD = 0.29, CI: [0.13, 0.45]), Provisc (SMD = 0.46, CI: [0.17, 0.76]), and Soft Shell (SMD = 0.58, CI: [0.30, 0.86]) was computed. On the other hand, results implied a nonsignificant increase in postoperative IOP with Healon GV (SMD = 0.07, CI: [−0.28, 0.41]), Healon5 (SMD = 0.15, CI: [−0.33, 0.64]), 2% HPMC (SMD = 0.32, CI: [−0.0, 0.64]), and OcuCoat (SMD = 0.26, CI: [−0.37, 0.9]). Further, a nonsignificant reduction in postoperative IOP was inferred with Viscoat + Provisc (SMD = −0.28, CI: [−2.23, 1.68]). Healon, Viscoat, and Soft Shell significantly increased IOP compared to a nonsignificant increase with Healon GV, Healon5, 2% HPMC, and OcuCoat, compared to a nonsignificant reduction in IOP with Viscoat + Provisc in 1-day follow-up. However, 2 studies evaluated Viscoat + Provisc indicating a need for more research. Results signified increase in postoperative IOP in 1-day follow-up with other OVDs (SMD = 1.62, CI: [0.5, 2.75]) (Figure 10).

**Figure 11:**
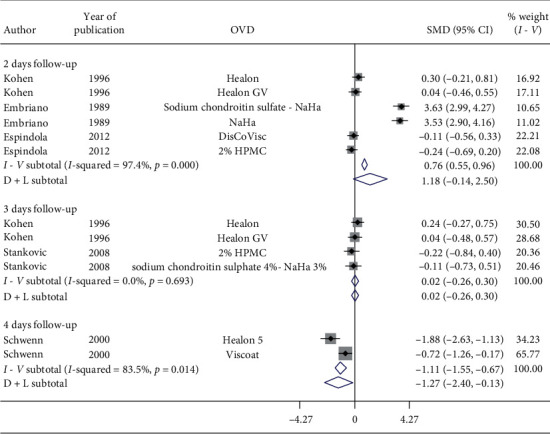
Forest plot for studies examining pre- and postoperative intraocular pressure (IOP) by two-, three-, and four-day follow-up.

Nonsignificant increase in postoperative IOP was observed in 2-day follow-up (SMD = 1.18, CI: [−0.14, 2.5]) and 3-day follow-up (SMD = 0.02, CI: [−0.26, 0.3]). Conversely, significant reduction in postoperative IOP was observed with Healon5 and Viscoat in 4-day follow-up (SMD = −1.27, CI: [−2.4, −0.13]) (Figure 11). However, 3 studies evaluated 2-day follow-up, 2 studies assessed 3-day follow-up, and 1 study considered 4-day follow-up; therefore, good quality RCTs with longer follow-ups are needed to make inferences.

**Figure 12:**
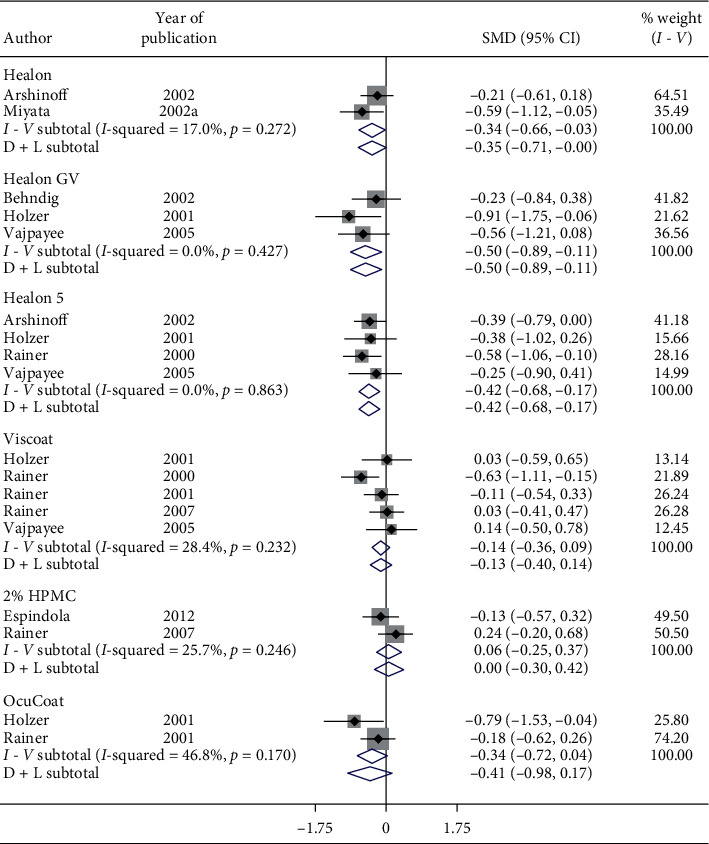
Forest plot for studies examining pre- and postoperative intraocular pressure (IOP) by one-week follow-up and ophthalmic viscoelastic devices (OVDs).

Meta-analysis showed nonsignificant reduction in postoperative IOP (Figure 12) in 1-week follow-up with Healon (SMD = −0.35, CI: [−0.71, 0.0]), Viscoat (SMD = −0.13, CI: [−0.4, 0.14]), 2% HPMC (SMD = 0.06, CI: [−0.3, 0.42]), and OcuCoat (SMD = −0.41, CI: [−0.98, 0.17]) compared to a significant increase in postoperative IOP in 1-week follow-up with Healon GV (SMD = −0.5, CI: [−0.89, −0.11]) and Healon5 (SMD = −0.42, CI: [−0.68, −0.17]). Thus, postoperative IOP in 1-week follow-up nonsignificantly decreases with Healon, Viscoat, 2% HPMC, and OcuCoat compared to a significant increase in IOP with Healon GV and Healon5.

**Figure 13:**
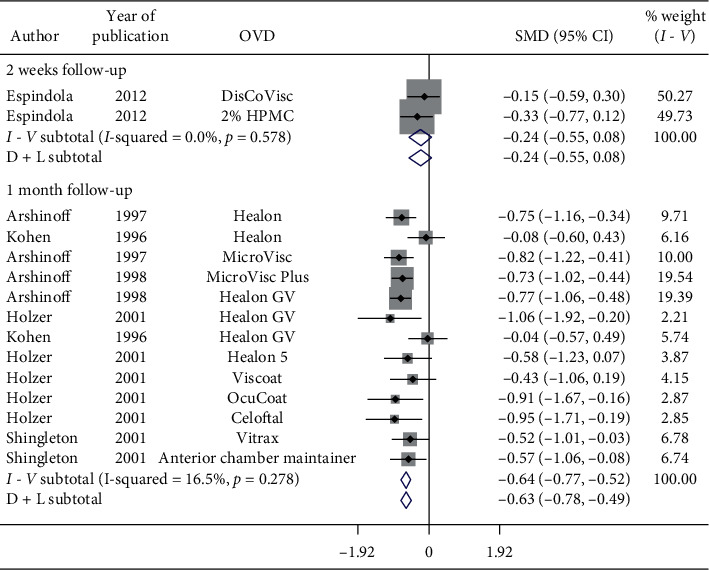
Forest plot for studies examining pre- and postoperative intraocular pressure (IOP) by two-week and one-month follow-up.

**Figure 14:**
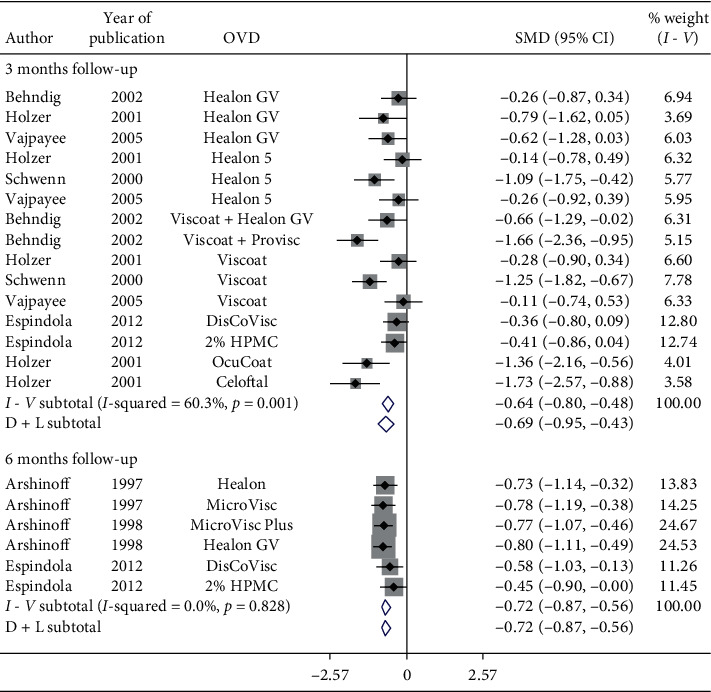
Forest plot for studies examining pre- and postoperative intraocular pressure (IOP) by three- and six-month follow-up.

Nonsignificant reduction in IOP occurred in 2-week follow-up (SMD = −0.24, CI: [−0.55, 0.08]) (Figure 13). However, significant reduction in postoperative IOP was observed in 1-month follow-up (SMD = −0.63, CI: [−0.78, −0.49]), 3-month follow-up (SMD = −0.69, CI: [−0.95, −0.43]), 6-month follow-up (SMD = −0.72, CI: [−0.87, −0.56]) (Figure 14) with various OVDs.

#### 3.3.2. Effect on Best Corrected Visual Acuity (BCVA)

**Figure 15:**
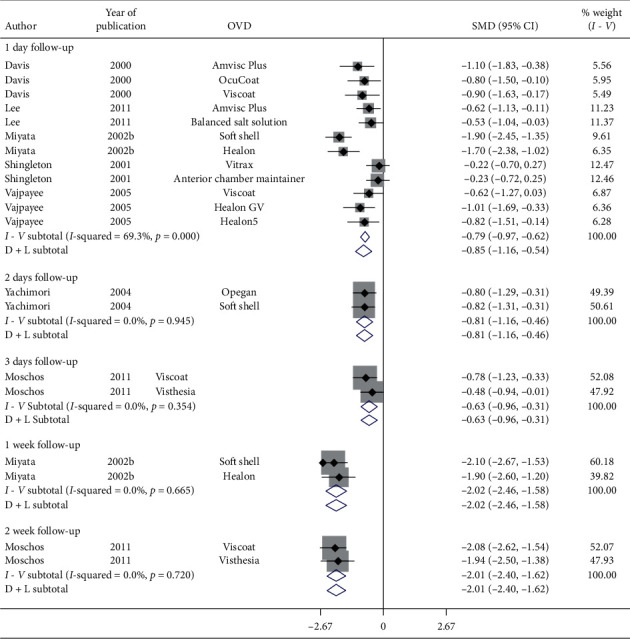
Forest plot for studies examining pre- and postoperative best corrected visual acuity (BCVA) by follow-up (days).

Figure 15 represents a forest plot of BCVA by follow-up (days) for articles evaluating various OVDs in patients with cataract. Significant heterogeneity between studies examining follow-up of 1-day (*I*^2^ = 69.3%) was observed. Results specified significant improvement in postoperative BCVA in 1-day follow-up (SMD = −0.85, CI: [−1.16, −0.54]), 2-day follow-up (SMD = −0.81, CI: [−1.16, −0.46]), 3-day follow-up (SMD = −0.63, CI: [−0.96, −0.31]), 7-day follow-up (SMD = −2.02, CI: [−2.46, −1.59]), and 14-day follow-up (SMD = −2.01, CI: [−2.4, −1.62]) irrespective of OVD used during cataract surgery. Therefore, BCVA improves within a day irrespective of the OVD utilized.

**Figure 16:**
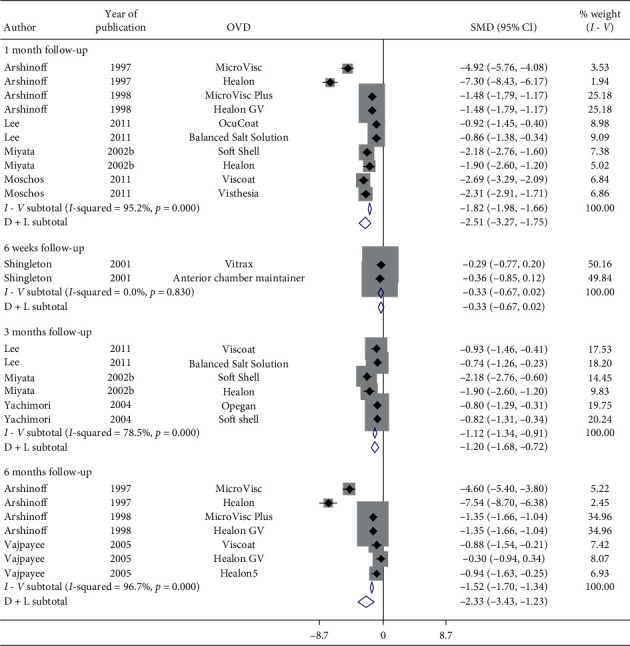
Forest plot for studies examining pre- and postoperative best corrected visual acuity (BCVA) follow-up (months).

Figure 16 presents a forest plot of BCVA by follow-up (months) for articles examining various OVDs in patients with cataract. Results implied significant improvement in postoperative BCVA in 1-month (SMD = −2.51, CI: [−3.27, −1.75]), 3-month (SMD = −1.20, CI: [−1.68, −0.72]), and 6-month follow-up (SMD = −2.33, CI: [−3.43, −1.23]). Irrespective of the OVDs used, postoperative BCVA does improve significantly. On the other hand, a single study showed a nonsignificant improvement in postoperative BCVA in 6-week follow-up (SMD = −0.33, CI: [−0.67, 0.02]) with Vitrax and Anterior Chamber Maintainer (ACM). Therefore, additional research is required.

## 4. Discussion

A systematic review was conducted to evaluate the effect of various OVDs in patients with cataract. A total of 36 RCTs (3893 subjects) were included for qualitative synthesis and 28 RCTs (2613 subjects) for quantitative synthesis. Percentage of reduction in IOP and standardized mean difference (SMD) in IOP as well as BCVA were computed. Meta-analysis results showed significant increase in postoperative IOP up to 5-hour follow-up irrespective of OVDs used. Therefore, removal of OVD is essential to avoid the IOP spikes. Results suggested a nonsignificant increase in postoperative IOP in 6-hour follow-up with Healon compared to a significant increase with Healon GV, Healon5, Viscoat, 2% HPMC, and OcuCoat.

Additionally, postoperative IOP significantly increases with Healon, Viscoat, and Soft Shell compared to a nonsignificant increase with Healon GV, Healon5, 2% HPMC, and OcuCoat, compared to a nonsignificant reduction with Viscoat + Provisc in 1-day follow-up. Postoperative IOP nonsignificantly decreases with Healon, Viscoat, 2% HPMC, and OcuCoat compared to a significant increase with Healon GV and Healon5 even after 1-week follow-up.

Meta-analysis results implied significant improvement in postoperative BCVA in a day regardless of OVD. However, a single study showed a nonsignificant improvement in postoperative BCVA in 6-week follow-up with Vitrax and Anterior Chamber Maintainer (ACM). Therefore, additional research is required.

The reason behind substantial between-study heterogeneity could reveal different study populations, demographics, inclusion/exclusion criteria, study location, design, OVDs used, surgeon's experience, available facilities to perform cataract surgery, rates of complications, and years when the surgeries were performed, as well as years when the studies were conducted. The results imply that good quality studies with longer follow-up periods need to be reported to better understand the optimal role of various OVDs in IOP management.

The limitations for systematic reviews and meta-analyses such as this one are necessary before conclusions are made. Firstly, included articles were of high, medium, and poor quality. However, few studies evaluating each OVD were available for analysis; all were included, irrespective of their quality. This is a recognized, but necessary, limitation due to the few clinical studies currently available examining each OVD. Secondly, meta-analysis of observational studies is influenced by inherent biases in the included articles [41]. For example, a multitude of other factors such as level of education, ethnicity, income status, socioeconomic status, previous ocular and nonocular surgeries, family history, other ocular and nonocular diseases, preoperative and postoperative medications, number of medications, and comorbidities (e.g., high blood pressure, diabetes, stroke, heart conditions, etc.) could influence the estimates in the original studies.

Our analysis indicated improvement in IOP with Viscoat + Provisc in 24-hour follow-up. Additionally, IOP nonsignificant upsurge was observed with Healon GV, Healon5, 2% HPMC, and OcuCoat compared to significant upsurge with Healon, Viscoat, and Soft Shell in 24-hour follow-up. The reason for this could be careful removal of OVD after cataract surgery. Therefore, it is not possible to differentiate OVD in increasing IOP. Further, additional research is needed to better understand how to maximize the utility of OVD in cataract management.

In conclusion, results indicated that postoperative IOP does significantly increase irrespective of OVD up to 5 hours of follow-up. Therefore, careful removal of OVD is essential to avoid the IOP spikes. More good quality RCTs are needed to better understand and define the position of OVDs in cataract management.

## Figures and Tables

**Table 1:** Characteristics of studies included in meta-analysis.

Author (year)	Study design	Study location	OVDs	*N* (eyes)	Mean age	Age (SD)
Arshinoff (1997) [11]	RCT	Canada	MicroVisc	51	70	10
	RCT	Canada	Healon	49	70	11

Arshinoff (1998) [4]	RCT	Canada	MicroVisc Plus	100	70	10
	RCT	Canada	Healon GV	100	66	10

Arshinoff (2002) [3]	RCT	Canada	Healon5	50	71.5	9.6
	RCT	Canada	Healon GV	99	71.7	9.6
	RCT	Canada	Healon	49	71.9	7.5

Auffarth (2017) [18]	RCT	Europe	Twinvisc	109	71.9	7.4
	RCT	Europe	DuoVisc	111	72.5	7.9

Behndig (2002) [19]	RCT	Sweden	Healon GV	21	72	10.4
	RCT	Sweden	Viscoat + Healon GV	20	72.8	11.7
	RCT	Sweden	Viscoat + Provisc	21	76.5	8.4

Chiselita (2008) [20]	RCT	Romania	Viscoat	44	68.8	9.8
	RCT	Romania	Provisc	52	68.8	9.8

Davis (2000) [2]	RCT	USA	Amvisc Plus	17	—	—
	RCT	USA	OcuCoat	17	—	—
	RCT	USA	Viscoat	16	—	—

Embriano (1989) [21]	RCT	USA	Sodium chondroitin sulfate-NaHa	50	—	—
	RCT	USA	NaHa	50	—	—

Espindola (2012) [22]	RCT	Brazil	DisCoVisc	39	71.5	7.9
	RCT	Brazil	2% HPMC	39	71.5	7.9

Holzer (2001) [1]	RCT	Germany	Healon GV	12	71.2	7.8
	RCT	Germany	Healon5	19	71.2	7.8
	RCT	Germany	Viscoat	20	71.2	7.8
	RCT	Germany	OcuCoat	15	71.2	7.8
	RCT	Germany	Celoftal	15	71.2	7.8

Hutz (1996) [23]	RCT	Germany	Methocel	50	—	—
	RCT	Germany	Viscoat	50	—	—
	RCT	Germany	Healon	50	—	—
	RCT	Germany	Healon GV	50	—	—

Kim (2004) [24]	RCT	Korea	Soft Shell (Viscoat + Hyal-2000)	69	64.15	12.92
	RCT	Korea	Viscoat	64	67.53	10.19
	RCT	Korea	Hyal-2000	64	63.22	11.51
	RCT	Korea	Provisc	55	63.3	12.78

Kocak-Altintas (2006) [25]	RCT	Turkey	BD Visc	83	65.6	11.1
	RCT	Turkey	Healon	83	65.8	11.3

Kohnen (1996) [12]	RCT	Germany	Healon	30	73.2	9.2
	RCT	Germany	Healon GV	30	73.2	9.2

Lee (2011) [26]	RCT	Korea	Amvisc Plus	31	65.42	12.20
	RCT	Korea	Balanced salt solution + Amvisc Plus	31	63.23	9.44

Miller (1999) [5]	RCT	USA	Healon GV	70	75.8	6.79
	RCT	USA	Viscoat	70	75.5	6.38

Miyata (2002a) [27]	RCT	Japan	Opegan	50	75.6	8.0
	RCT	Japan	Healon	28	74.3	8.7

Miyata (2002b) [28]	RCT	Japan	Soft Shell	37	74.8	10.2
	RCT	Japan	Healon	23	76.5	8.5

Moschos (2011) [13]	RCT	Greece	Viscoat	41	77.6	8.4
	RCT	Greece	Visthesia	36	77.7	8.7

Neumayer (2008) [29]	RCT	UK	Neocrom Cohesive	29	75	—
	RCT	UK	Healon	29	75	—

Oshika (2004) [30]	RCT	Japan	Healon5	79	69	10
	RCT	Japan	Healon	78	71	9

Oshika (2010) [6]	RCT	Japan	DisCoVisc	157	70.3	8.2
	RCT	Japan	Healon5	166	70.3	7.9

Ray-Chaudhary (2005) [31]	RCT	UK	Ophthalin	51	—	—
	RCT	UK	HPMC-Ophtal	50	—	—

Rainer (2000) [7]	RCT	Austria	Healon5	35	75.5	9.1
	RCT	Austria	Viscoat	35	75.5	9.1

Rainer (2001) [8]	RCT	Austria	OcuCoat	40	75.9	9.3
	RCT	Austria	Viscoat	40	75.9	9.3

Rainer (2007) [10]	RCT	Austria	NaHa 1%	40	75.1	8.0
	RCT	Austria	2% HPMC	40	75.1	8.0

Rainer (2008) [32]	RCT	Austria	Viscoat	30	76.6	7.4
	RCT	Austria	DuoVisc	30	76.6	7.4

Ravalico (1997) [33]	RCT	Italy	Healon	16	64.06	5.97
	RCT	Italy	Healon GV	15	61.64	9.56
	RCT	Italy	Viscoat	14	62.67	6.34
	RCT	Italy	Hymecel	13	62.85	7.55

Schwenn (2000) [34]	RCT	Germany	Healon5	20	—	—
	RCT	Germany	Viscoat	28	—	—

Stankovic (2008) [35]	RCT	Serbia	2% HPMC	20	—	—
	RCT	Serbia	Chondroitin sulfate 4%- NaHa 3%	20	—	—

Storr-Paulsen (2007) [36]	RCT	Denmark	Celoftal	17	77.9	8.1
	RCT	Denmark	Vitrax	16	76.6	10.4
	RCT	Denmark	Healon	19	76.4	13.1

Strobel (1997) [37]	RCT	Germany	Healon GV	30	68.9	10.8
	RCT	Germany	Healon	30	73.6	10.2

Thirumalai (2007) [38]	RCT	UK	Healon GV	415	—	—

Vajpayee (2005) [39]	RCT	India	Viscoat	19	69.6	9.2
	RCT	India	Healon GV	19	65.8	7.8
	RCT	India	Healon5	18	70.8	9.9

Yachimori (2004) [40]	RCT	Japan	Opegan	34	68.6	8.2
	RCT	Japan	Soft Shell	35	70.7	8.3

**Table 2:** Reported wash-out times for OVDs.

Author (year)	OVDs	*N* (eyes)	Wash-out time for OVD (seconds)
			Mean [SD]
Espindola (2012)	DisCoVisc	39	10.2 [3.6]
	2% HPMC	39	13.2 [5.4]

Hutz (1996)	Methocel	50	Healon was the easiest and quickest to remove from the anterior chamber. Healon GV was also removed easily in a short time; however, in two patients very small particles of the iris pigment were mobilized by the Healon GV. Visco adhered to the intraocular structures and was difficult to remove from the eye; Methocel was difficult to remove from the corneal endothelium
	Viscoat	50	
	Healon	50	
	Healon GV	50	

Kim (2004)	Soft Shell (Viscoat + Hyal-2000)	69	Soft Shell technique enhances OVD removal at the conclusion of surgery
	Viscoat	64	—
	Hyal-2000	64	—
	Provisc	55	—

Kohnen (1996)	Healon	30	No difference between the two groups with 20-second and 40-second wash-out times
	Healon GV	30	

Lee (2011)	Amvisc Plus	31	50.42 [3.83]
	Balanced salt Solution + Amvisc Plus	31	8.29 [4.40]

Miller (1999)	Healon GV	70	19.8 [22.2]
	Viscoat	70	75 [16.8]

Oshika (2004)	Healon5	78	Healon was significantly easier to remove compared to Healon5
	Healon	79	

Oshika (2010)	DisCoVisc	154	DisCoVisc showed significantly better performance than Healon5 in terms of removal
	Healon5	163	

Rainer (2007)	NaHa 1%	40	Removal of NaHa 1% was easy and faster than 2% HPMC in bulk fashion
	2% HPMC	40	

Vajpayee (2005)	Viscoat	19	66.6 [11.2]
	Healon GV	19	45.1 [9.0]
	Healon5	18	55.47 [6.6]

**Table 3:** Complication rates reported for OVDs.

Author (year)	OVDs	*N* (eyes)	Adverse events (rates in %)
Auffarth (2017)	Twinvisc	109	Ocular hypertension (12.6%), corneal edema (0.9%), cystoid macular edema (0.9%); IOP ≥ 30 mmHg in 6 hours postop (6.5%), 24 hours (0.9%), 7 days (0.9%), 30 days (0%), 90 days (0%)

	DuoVisc	111	Ocular hypertension (17.6%), corneal edema (0.9%), inflammation (0.9%), capsule break (0.9%), bubbles in Viscoat OVD (0.9%); IOP ≥ 30 mmHg in 6 hours postop (7.2%), 24 hours (0%), 7 days (0%), 30 days (0%), 90 days (0%)

Espindola (2012)	DisCoVisc	39	No intraoperative and postoperative complications
	2% HPMC	39	

Hutz (1996)	Methocel	50	Pressure peaks up to 44 mm·Hg requiring acetazolamide (Diamox) treatment occurred twice in the Healon GV group and once in the Healon group postoperatively. Peaks up to 38 mm·Hg occurred three times in the Viscoat group, and peaks up to 35 mm Hg occurred six times in the Methocel group
	Viscoat	50	
	Healon	50	
	Healon GV	50	

Oshika (2004)	Healon5	78	IOP elevation (5.1%), corneal edema (2.5%), nausea and vomiting (1.3%)
	Healon	79	IOP elevation (1.3%), corneal edema (1.3%)

Oshika (2010)	DisCoVisc	154	IOP ≥ 30 mmHg in 5 h postop (7.2%)
	Healon5	163	IOP ≥ 30 mmHg in 5 h postop (7.4%), mild corneal edema (0.6%), macular edema (0.6%)

Ray-Chaudhary (2006)	Ophthalin	51	No significant difference in the number of complications between the two groups
	HPMC-Ophtal	52	

Rainer (2007)	NaHa 1%	40	IOP ≥ 30 mmHg in 30 mins postop (3%), 1 hour postop (5%), 2 hours postop (3%), 3 hours postop (5%), 4 hours postop (8%), 6 hours postop (13%), 8 hours postop (8%), 20–24 hours postop (0%)
	2% HPMC	40	IOP ≥ 30 mmHg in 30 mins postop (8%), 1 hour postop (13%), 2 hours postop (23%), 3 hours postop (13%), 4 hours postop (8%), 6 hours postop (10%), 8 hours postop (10%), 20–24 hours postop (0%)

Thirumalai (2007)	Healon GV	415	In 2 hours follow-up: IOP ≥ 30 mmHg (7.2%), IOP ≥35 mmHg (4%). In 24-hour follow-up: IOP ≥ 30 mmHg (8.8%), IOP ≥ 35 mmHg (4%). In 2-day follow-up: IOP ≥30 mmHg (7%), IOP ≥ 35 mmHg (3.5%)

## Data Availability

The data used to support the findings of the study are available from the corresponding author upon request.

## Supplementary Materials

Supplementary Materials S1: PRISMA 2009 checklist. S2: search strategy for EMBASE and MEDLINE. S3: Levels 1, 2, and 3 screening questions. S4: Funnel plots. S5: Intraocular pressure (IOP) reported in studies included in the meta-analysis.
