# Supplementary material for: Impact of Ophthalmic Viscosurgical Devices in Cataract Surgery
Source: J Ophthalmol. 2020 Oct 20;2020:7801093. doi: 10.1155/2020/7801093 (PMC7593745; doi:10.1155/2020/7801093)
Supplement: Supplementary Materials — S1: PRISMA 2009 checklist. S2: search strategy for EMBASE and MEDLINE. S3: Levels 1, 2, and 3 screening questions. S4: Funnel plots. S5: Intraocular pressure (IOP) reported in studies included in the meta-analysis. [file 7801093.f1.doc]

| **Section/topic** | **#** | **Checklist item** | **Reported on page #** |
| --- | --- | --- | --- |
| **TITLE** | | |  |
| Title | 1 | Identify the report as a systematic review, meta-analysis, or both. | 1 |
| **ABSTRACT** | | |  |
| Structured summary | 2 | Provide a structured summary including, as applicable: background; objectives; data sources; study eligibility criteria, participants, and interventions; study appraisal and synthesis methods; results; limitations; conclusions and implications of key findings; systematic review registration number. | 2 |
| **INTRODUCTION** | | |  |
| Rationale | 3 | Describe the rationale for the review in the context of what is already known. | 3 |
| Objectives | 4 | Provide an explicit statement of questions being addressed with reference to participants, interventions, comparisons, outcomes, and study design (PICOS). | 4 |
| **METHODS** | | |  |
| Protocol and registration | 5 | Indicate if a review protocol exists, if and where it can be accessed (e.g., Web address), and, if available, provide registration information including registration number. | 4 |
| Eligibility criteria | 6 | Specify study characteristics (e.g., PICOS, length of follow-up) and report characteristics (e.g., years considered, language, publication status) used as criteria for eligibility, giving rationale. | 5 |
| Information sources | 7 | Describe all information sources (e.g., databases with dates of coverage, contact with study authors to identify additional studies) in the search and date last searched. | 4, 5 |
| Search | 8 | Present full electronic search strategy for at least one database, including any limits used, such that it could be repeated. | 5, S2 Supplementary File |
| Study selection | 9 | State the process for selecting studies (i.e., screening, eligibility, included in systematic review, and, if applicable, included in the meta-analysis). | 6, S3 Supplementary File |
| Data collection process | 10 | Describe method of data extraction from reports (e.g., piloted forms, independently, in duplicate) and any processes for obtaining and confirming data from investigators. | 6 |
| Data items | 11 | List and define all variables for which data were sought (e.g., PICOS, funding sources) and any assumptions and simplifications made. | 6 |
| Risk of bias in individual studies | 12 | Describe methods used for assessing risk of bias of individual studies (including specification of whether this was done at the study or outcome level), and how this information is to be used in any data synthesis. | 6 |
| Summary measures | 13 | State the principal summary measures (e.g., risk ratio, difference in means). | 7 |
| Synthesis of results | 14 | Describe the methods of handling data and combining results of studies, if done, including measures of consistency (e.g., I^2^) for each meta-analysis. | 7, 8 |

Page 1 of 2

| **Section/topic** | **#** | **Checklist item** | **Reported on page #** |
| --- | --- | --- | --- |
| Risk of bias across studies | 15 | Specify any assessment of risk of bias that may affect the cumulative evidence (e.g., publication bias, selective reporting within studies). | 6 |
| Additional analyses | 16 | Describe methods of additional analyses (e.g., sensitivity or subgroup analyses, meta-regression), if done, indicating which were pre-specified. | 7, 8 |
| **RESULTS** | | |  |
| Study selection | 17 | Give numbers of studies screened, assessed for eligibility, and included in the review, with reasons for exclusions at each stage, ideally with a flow diagram. | 8 |
| Study characteristics | 18 | For each study, present characteristics for which data were extracted (e.g., study size, PICOS, follow-up period) and provide the citations. | 8 |
| Risk of bias within studies | 19 | Present data on risk of bias of each study and, if available, any outcome level assessment (see item 12). | 9 |
| Results of individual studies | 20 | For all outcomes considered (benefits or harms), present, for each study: (a) simple summary data for each intervention group (b) effect estimates and confidence intervals, ideally with a forest plot. | 10 |
| Synthesis of results | 21 | Present results of each meta-analysis done, including confidence intervals and measures of consistency. | 11, 12, 13 |
| Risk of bias across studies | 22 | Present results of any assessment of risk of bias across studies (see Item 15). | 9 |
| Additional analysis | 23 | Give results of additional analyses, if done (e.g., sensitivity or subgroup analyses, meta-regression [see Item 16]). | 11, 12, 13 |
| **DISCUSSION** | | |  |
| Summary of evidence | 24 | Summarize the main findings including the strength of evidence for each main outcome; consider their relevance to key groups (e.g., healthcare providers, users, and policy makers). | 13, 14 |
| Limitations | 25 | Discuss limitations at study and outcome level (e.g., risk of bias), and at review-level (e.g., incomplete retrieval of identified research, reporting bias). | 14, 15 |
| Conclusions | 26 | Provide a general interpretation of the results in the context of other evidence, and implications for future research. | 15 |
| **FUNDING** | | |  |
| Funding | 27 | Describe sources of funding for the systematic review and other support (e.g., supply of data); role of funders for the systematic review. | 15, Cover letter |

*From:*  Moher D, Liberati A, Tetzlaff J, Altman DG, The PRISMA Group (2009). Preferred Reporting Items for Systematic Reviews and Meta-Analyses: The PRISMA Statement. PLoS Med 6(6): e1000097. doi:10.1371/journal.pmed1000097

For more information, visit: **www.prisma-statement.org**.

Page 2 of 2

**S2: SEARCH STRATEGY for EMBASE and MEDLINE**

**1. MEDLINE – January 14, 2019**

1. exp Viscoelastic Substances/

2. Cataract Extraction/

3. ophthalmic viscosurgical device*.mp. [mp=title, abstract, original title, name of substance word, subject heading word, keyword heading word, protocol supplementary concept word, rare disease supplementary concept word, unique identifier]

4. ophthalmic viscoelastic device*.mp. [mp=title, abstract, original title, name of substance word, subject heading word, keyword heading word, protocol supplementary concept word, rare disease supplementary concept word, unique identifier]

5. viscoadaptive*.mp. [mp=title, abstract, original title, name of substance word, subject heading word, keyword heading word, protocol supplementary concept word, rare disease supplementary concept word, unique identifier]

6. hyaluronic acid.mp. [mp=title, abstract, original title, name of substance word, subject heading word, keyword heading word, protocol supplementary concept word, rare disease supplementary concept word, unique identifier]

7. Hyaluronic Acid/

8. Chondroitin/

9. chondroitin.mp. [mp=title, abstract, original title, name of substance word, subject heading word, keyword heading word, protocol supplementary concept word, rare disease supplementary concept word, unique identifier]

10. hydroxypropylmethylcellulose.mp. [mp=title, abstract, original title, name of substance word, subject heading word, keyword heading word, protocol supplementary concept word, rare disease supplementary concept word, unique identifier]

11. 1 or 3 or 4 or 5 or 6 or 7 or 8 or 9 or 10

12. 2 and 11

13. limit 12 to english language

14. limit 13 to humans

Number of articles retrieved from MEDLINE: 213

**2. EMBASE – January 14, 2019**

1. exp ophthalmic viscosurgical device/

2. hyaluronic acid/

3. exp cataract extraction/

4. chondroitin sulfate/

5. ophthalmic viscosurgical device*.mp. [mp=title, abstract, subject headings, heading word, drug trade name, original title, device manufacturer, drug manufacturer, device trade name, keyword]

6. ophthalmic viscoelastic device*.mp. [mp=title, abstract, subject headings, heading word, drug trade name, original title, device manufacturer, drug manufacturer, device trade name, keyword]

7. viscoadaptive*.mp. [mp=title, abstract, subject headings, heading word, drug trade name, original title, device manufacturer, drug manufacturer, device trade name, keyword]

8. hydroxypropylmethylcellulose/

9. hyaluronic acid.mp. [mp=title, abstract, subject headings, heading word, drug trade name, original title, device manufacturer, drug manufacturer, device trade name, keyword]

10. chondroitin.mp. [mp=title, abstract, subject headings, heading word, drug trade name, original title, device manufacturer, drug manufacturer, device trade name, keyword]

11. hydroxypropylmethylcellulose.mp. [mp=title, abstract, subject headings, heading word, drug trade name, original title, device manufacturer, drug manufacturer, device trade name, keyword]

12. 1 or 2 or 4 or 5 or 6 or 7 or 8 or 9 or 10 or 11

13. 3 and 12

14. limit 13 to english language

15. limit 14 to human

Number of articles retrieved from EMBASE: 639

**S3: LEVEL 1, 2, AND 3 SCREENING QUESTIONS**

**Level 1 (Title) Screening**

1. Does the study look at the use of ophthalmic viscosurgical device (OVD)?
   1. Yes b. No c. Unclear
2. Is this a research study or review article (not an editorial, opinion or case report)?
   1. Yes b. No c. Unclear
3. Is the study published in 2000 or later?
   1. Yes b. No c. Unclear

**Level 2 (Abstract) Screening**

1. Does the study look at complication(s) of OVD (eg IOP spikes, endothelial cell loss) or ease of surgery (eg ease of use, maintenance of anterior chamber) or cost?
   1. Yes b. No c. Unclear
2. Did patients in this study undergo cataract surgery?
   1. Yes b. No c. Unclear
3. Does the study consist of a sample size of 20 or more patients?
   1. Yes b. No c. Unclear
4. Are the patients in this study adults (ie not pediatric population)?
   1. Yes b. No c. Unclear
5. Is this a research study (not a pilot study)?
   1. Yes b. No c. Unclear

**Level 3 (Full-text) Screening**

1. Are patients enrolled in this study healthy ocular-wise (ie. has no other ocular co-morbidities)?
   1. Yes b. No c. Unclear
2. Did patients receive only phacoemulsification cataract surgery (ie no other ocular surgery/procedure)?
   1. Yes b. No c. Unclear
3. Does the study explicitly discuss the post-operative outcomes including intraocular pressure, or best corrected visual acuity, or central corneal thickness, or endothelial cell loss/density?
   1. Yes b. No c. Unclear

**S4: FUNNEL PLOTS**

Figure 1.2: Funnel Plot for Studies Examining Pre- and Post-Operative Intraocular Pressure

(IOP).


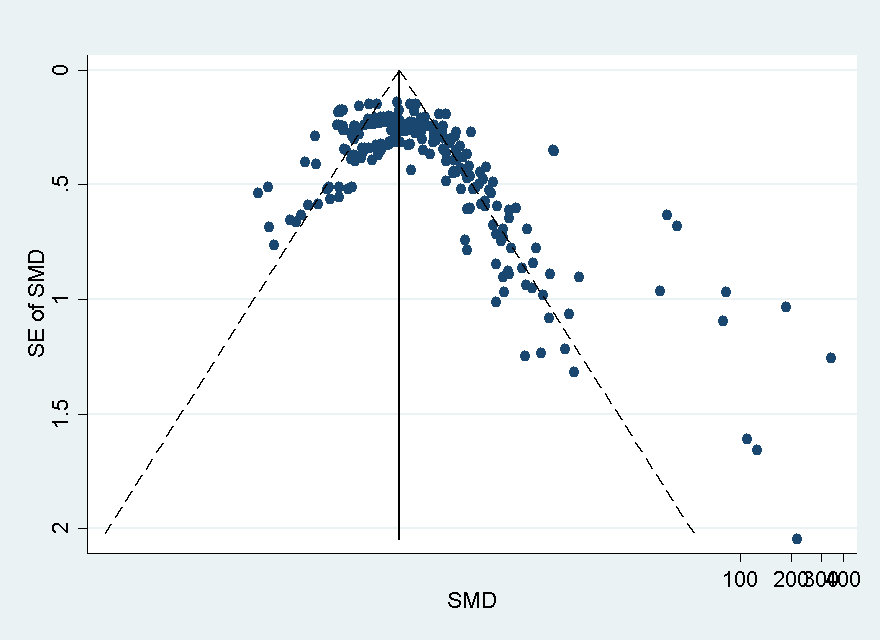


Figure 1.3: Funnel Plot For Studies Examining Pre- And Post-Operative Best Corrected Visual

Acuity (BCVA).


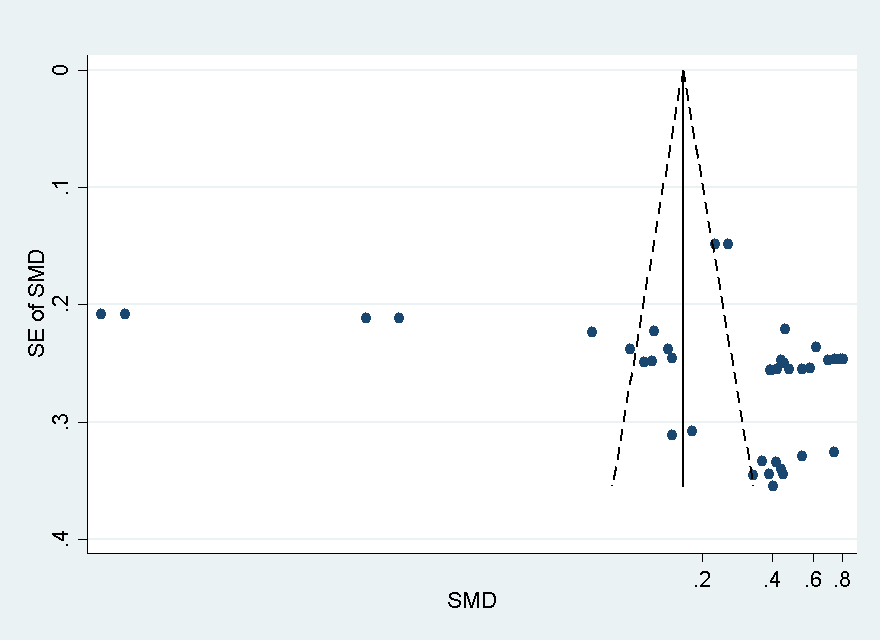


**S5: INTRAOCULAR PRESSURE (IOP) REPORTED IN STUDIES INCLUDED IN META-ANALYSIS**

| **Author (Year)** | **OVDs** | **N (eyes)** | **Follow-up** | **Mean IOP** | **IOP (SD)** | **Mean Difference (SD)** |
| --- | --- | --- | --- | --- | --- | --- |
| Arshinoff (1997) | MicroVisc | 51 | Preop | 16 | 2.4 | - |
|  | MicroVisc | 9 | 6 hours | 13 | 6.5 |  |
|  | MicroVisc | 51 | 24 hours | 15 | 4.3 |  |
|  | MicroVisc | 51 | 5 days | 13 | 3.0 |  |
|  | MicroVisc | 46 | 1 month | 14 | 2.5 |  |
|  | MicroVisc | 41 | 6 months | 14 | 2.7 |  |
|  | Healon | 49 | Preop | 15 | 2.7 |  |
|  | Healon | 12 | 6 hours | 13 | 4.2 |  |
|  | Healon | 48 | 24 hours | 15 | 4.2 |  |
|  | Healon | 48 | 5 days | 13 | 2.8 |  |
|  | Healon | 46 | 1 month | 13 | 2.6 |  |
|  | Healon | 41 | 6 months | 13 | 2.8 |  |
| Arshinoff (1998) | MicroVisc Plus | 50 | Preop | 16 | 2.7 |  |
|  | MicroVisc Plus | 14 | 6 hours | 15 | 4.4 |  |
|  | MicroVisc Plus | 48 | 24 hours | 17 | 5 |  |
|  | MicroVisc Plus | 48 | 5 days | 15 | 2.5 |  |
|  | MicroVisc Plus | 49 | 1 month | 14 | 2.8 |  |
|  | MicroVisc Plus | 43 | 6 months | 14 | 2.5 |  |
|  | Healon GV | 50 | Preop | 16 | 2.6 |  |
|  | Healon GV | 19 | 6 hours | 17 | 7.8 |  |
|  | Healon GV | 50 | 24 hours | 16 | 3.8 |  |
|  | Healon GV | 50 | 5 days | 14 | 2.8 |  |
|  | Healon GV | 49 | 1 month | 14 | 2.6 |  |
|  | Healon GV | 45 | 6 months | 14 | 2.4 |  |
| Arshinoff (2002) | Healon5 | 50 | 4-6 hours | 15.6 | 2.4 | 18.1 |
|  | Healon5 | 50 | 24 hours | 15.6 | 2.4 | 19.4 |
|  | Healon5 | 50 | 1 week | 15.6 | 2.4 | 14.4 |
|  | Healon GV vs Healon5 | 50 | 4-6 hours | 15.6 | 2.2 | 18.2 |
|  | Healon GV | 50 | 24 hours | 15.6 | 2.2 | 20.2 |
|  | Healon GV | 50 | 1 week | 15.6 | 2.2 | 14.1 |
|  | Healon | 49 | 4-6 hours | 15 | 2.4 | 17.6 |
|  | Healon | 49 | 24 hours | 15 | 2.4 | 17.9 |
|  | Healon | 49 | 1 week | 15 | 2.4 | 14.4 |

| **Author (Year)** | **OVDs** | **N (eyes)** | **Follow-up** | **Mean Pre-op IOP** | **Pre-op IOP (SD)** | **Post-op IOP**  **Mean** | **Post-op IOP (SD)** | **Mean Difference (SD)** |
| --- | --- | --- | --- | --- | --- | --- | --- | --- |
| Auffarth (2017) | Twinvisc | 109 | 6 hours | 15.18 | 2.72 | 19.04 | 6.68 | 3.9 (6.5) |
|  | Twinvisc | 109 | 24 hours | 15.18 | 2.72 | 15.15 | 4.58 | 0.0 (4.5) |
|  | Twinvisc | 109 | 1 week | 15.18 | 2.72 | 14.58 | 4.43 | -0.6 (1.7) |
|  | Twinvisc | 109 | 4 weeks | 15.18 | 2.72 | 13.51 | 2.89 | -1.7 (3.0) |
|  | Twinvisc | 109 | 12 weeks | 15.18 | 2.72 | 12.74 | 2.81 | -2.4 (3.3) |
|  | Duovisc | 111 | 6 hours | 15.26 | 2.4 | 20.21 | 6.55 | 5.05 (6.1) |
|  | Duovisc | 111 | 24 hours | 15.26 | 2.4 | 15.95 | 4.56 | 0.7 (4.1) |
|  | Duovisc | 111 | 1 week | 15.26 | 2.4 | 14.25 | 2.79 | -1.0 (2.6) |
|  | Duovisc | 111 | 4 weeks | 15.26 | 2.4 | 13.23 | 2.99 | -2.0 (3.1) |
|  | Duovisc | 111 | 12 weeks | 15.26 | 2.4 | 13.23 | 2.46 | 2.5 (2.5) |
| Behndig (2002) | Healon GV | 21 | 5 hours | 17.8 | 9.3 | 17.8 | 4.7 | 0.0 (4.6) |
|  | Healon GV | 21 | 24 hours | 17.8 | 9.3 | 17.3 | 4 | -0.5 (2.5) |
|  | Healon GV | 21 | 1 week | 17.8 | 9.3 | 16.2 | 3.3 | -1.6 (2.5) |
|  | Healon GV | 21 | 1 month | 17.8 | 9.3 | 15.9 | 4.1 | -2.0 (3.2) |
|  | Viscoat + Healon GV | 20 | 5 hours | 18.8 | 7.5 | 17.3 | 3.5 | 1.5 (4.0) |
|  | Viscoat + Healon GV | 20 | 24 hours | 18.8 | 7.5 | 16.7 | 4.6 | -0.4 (4.0) |
|  | Viscoat + Healon GV | 20 | 1 week | 18.8 | 7.5 | 15.2 | 3.8 | -0.9 (4.5) |
|  | Viscoat + Healon GV | 20 | 1 month | 18.8 | 7.5 | 15.1 | 2.7 | -0.7 (4.0) |
|  | Viscoat + Provisc | 21 | 5 hours | 21.1 | 4.1 | 19.4 | 3.3 | 1.7 (2.6) |
|  | Viscoat + Provisc | 21 | 24 hours | 21.1 | 4.1 | 16.0 | 3.8 | -0.9 (4.2) |
|  | Viscoat + Provisc | 21 | 1 week | 21.1 | 4.1 | 15.8 | 3.2 | -1.1 (3.1) |
|  | Viscoat + Provisc | 21 | 1 month | 21.1 | 4.1 | 14.7 | 3.6 | -2.2 (3.3) |

| **Author (Year)** | **OVDs** | **N (eyes)** | **Follow-up** | **Mean Pre-operative IOP** | **Pre-operative IOP (SD)** | **Post-operative IOP**  **Mean** | **Post-operative IOP (SD)** | **Mean Difference (SD)** |
| --- | --- | --- | --- | --- | --- | --- | --- | --- |
| Chiselita (2008) | Viscoat | 44 | 1 hour | 14.59 | 3.11 | 26.08 | 10.38 |  |
|  | Viscoat | 44 | 2 hours | 14.59 | 3.11 | 31.83 | 9.78 |  |
|  | Viscoat | 44 | 3 hours | 14.59 | 3.11 | 30.49 | 9.46 |  |
|  | Viscoat | 44 | 6 hours | 14.59 | 3.11 | 23.83 | 8.67 |  |
|  | Viscoat | 44 | 9 hours | 14.59 | 3.11 | 21.66 | 7.88 |  |
|  | Viscoat | 44 | 24 hours | 14.59 | 3.11 | 17.02 | 5.68 |  |
|  | Provisc | 52 | 1 hour | 13.79 | 2.88 | 19.83 | 8.21 |  |
|  | Provisc | 52 | 2 hours | 13.79 | 2.88 | 23.5 | 6.78 |  |
|  | Provisc | 52 | 3 hours | 13.79 | 2.88 | 23.42 | 7.28 |  |
|  | Provisc | 52 | 6 hours | 13.79 | 2.88 | 22.13 | 7.26 |  |
|  | Provisc | 52 | 9 hours | 13.79 | 2.88 | 19.24 | 5.76 |  |
|  | Provisc | 52 | 24 hours | 13.79 | 2.88 | 15.97 | 4.06 |  |
|  | Viscoat + Provisc | 51 | 1 hour | 14.38 | 2.92 | 20.02 | 10.1 |  |
|  | Viscoat + Provisc | 51 | 2 hours | 14.38 | 2.92 | 24.65 | 9.94 |  |
|  | Viscoat + Provisc | 51 | 3 hours | 14.38 | 2.92 | 26.38 | 9.71 |  |
|  | Viscoat + Provisc | 51 | 6 hours | 14.38 | 2.92 | 25.1 | 9.42 |  |
|  | Viscoat + Provisc | 51 | 9 hours | 14.38 | 2.92 | 22.4 | 7.96 |  |
|  | Viscoat + Provisc | 51 | 24 hours | 14.38 | 2.92 | 17.7 | 6.02 |  |
| Embriano (1989) | Sodium chondroitin sulfate - NaHa | 50 | 2 hours | 18.0 | 0.05 | 22.14 | 2.77 |  |
|  | Sodium chondroitin sulfate - NaHa | 50 | 4 hours | 18.0 | 0.05 | 27.2 | 3.46 |  |
|  | Sodium chondroitin sulfate - NaHa | 50 | 8 hours | 18.0 | 0.05 | 33.24 | 4.91 |  |
|  | Sodium chondroitin sulfate - NaHa | 50 | 16 hours | 18.0 | 0.05 | 35.54 | 4.25 |  |

| **Author (Year)** | **OVDs** | **N (eyes)** | **Follow-up** | **Mean Pre-operative IOP** | **Pre-operative IOP (SD)** | **Post-operative IOP**  **Mean** | **Post-operative IOP (SD)** | **IOPR% [SD_IOPR%_]** |
| --- | --- | --- | --- | --- | --- | --- | --- | --- |
| Embriano (1989) | Sodium chondroitin sulfate - NaHa | 50 | 24 hours | 18.0 | 0.05 | 32.38 | 3.89 |  |
|  | Sodium chondroitin sulfate - NaHa | 50 | 48 hours | 18.0 | 0.05 | 26.54 | 3.33 |  |
|  | NaHa | 50 | 2 hours | 18.0 | 0.05 | 22.04 | 2.73 |  |
|  | NaHa | 50 | 4 hours | 18.0 | 0.05 | 31.44 | 4.29 |  |
|  | NaHa | 50 | 8 hours | 18.0 | 0.05 | 40.6 | 6.8 |  |
|  | NaHa | 50 | 16 hours | 18.0 | 0.05 | 46.82 | 7.57 |  |
|  | NaHa | 50 | 24 hours | 18.0 | 0.05 | 41.3 | 6.81 |  |
|  | NaHa | 50 | 48 hours | 18.0 | 0.05 | 31.38 | 5.36 |  |
| Espindola (2012) | DisCoVisc | 39 | 5 hours | 13.4 | 2.3 | 14.3 | 3.6 |  |
|  | DisCoVisc | 39 | 24 hours | 13.4 | 2.3 | 13.5 | 3.9 |  |
|  | DisCoVisc | 39 | 48 hours | 13.4 | 2.3 | 13.1 | 3.0 |  |
|  | DisCoVisc | 39 | 1 week | 13.4 | 2.3 | 12.9 | 2.4 |  |
|  | DisCoVisc | 39 | 2 weeks | 13.4 | 2.3 | 13.0 | 3.1 |  |
|  | DisCoVisc | 39 | 3 months | 13.4 | 2.3 | 12.6 | 2.2 |  |
|  | DisCoVisc | 39 | 6 months | 13.4 | 2.3 | 12.2 | 1.8 |  |
|  | 2% HPMC | 39 | 5 hours | 13.5 | 2.1 | 14.4 | 3.8 |  |
|  | 2% HPMC | 39 | 24 hours | 13.5 | 2.1 | 14.3 | 3.6 |  |
|  | 2% HPMC | 39 | 48 hours | 13.5 | 2.1 | 12.9 | 2.8 |  |
|  | 2% HPMC | 39 | 1 week | 13.5 | 2.1 | 13.1 | 3.9 |  |
|  | 2% HPMC | 39 | 2 weeks | 13.5 | 2.1 | 12.6 | 3.3 |  |
|  | 2% HPMC | 39 | 3 months | 13.5 | 2.1 | 12.6 | 2.3 |  |
|  | 2% HPMC | 39 | 6 months | 13.5 | 2.1 | 12.5 | 2.3 |  |
| Holzer (2001) | Healon GV | 12 | 6 hours | 16 | 3.3 | 21.6 | 4.5 |  |
|  | Healon GV | 12 | 24 hours | 16 | 3.3 | 17.1 | 7.8 |  |
|  | Healon GV | 12 | 1 week | 16 | 3.3 | 13.3 | 2.6 |  |
|  | Healon GV | 12 | 1 month | 16 | 3.3 | 13.1 | 2 |  |
|  | Healon GV | 12 | 3 months | 16 | 3.3 | 13.4 | 3.3 |  |
|  | Healon5 | 19 | 6 hours | 14.5 | 2.7 | 24.9 | 7.1 |  |
|  | Healon5 | 19 | 24 hours | 14.5 | 2.7 | 15.7 | 4.1 |  |
|  | Healon5 | 19 | 1 week | 14.5 | 2.7 | 13.5 | 2.6 |  |
|  | Healon5 | 19 | 1 month | 14.5 | 2.7 | 12.9 | 2.8 |  |
|  | Healon5 | 19 | 3 months | 14.5 | 2.7 | 14.1 | 2.9 |  |
|  | Viscoat | 20 | 6 hours | 15.4 | 3 | 23.6 | 7.5 |  |
|  | Viscoat | 20 | 24 hours | 15.4 | 3 | 17.1 | 4.5 |  |

| **Author (Year)** | **OVDs** | **N (eyes)** | **Follow-up** | **Mean Pre-op IOP** | **Pre-op IOP (SD)** | **Post-op IOP**  **Mean** | **Post-op IOP (SD)** | **IOPR% [SD_IOPR%_]** |
| --- | --- | --- | --- | --- | --- | --- | --- | --- |
|  | Viscoat | 20 | 1 week | 15.4 | 3 | 15.5 | 3.5 |  |
|  | Viscoat | 20 | 1 month | 15.4 | 3 | 14.2 | 2.5 |  |
|  | Viscoat | 20 | 3 months | 15.4 | 3 | 14.6 | 2.7 |  |
|  | OcuCoat | 15 | 6 hours | 16.7 | 3.3 | 22.1 | 7.4 |  |
|  | OcuCoat | 15 | 24 hours | 16.7 | 3.3 | 19.2 | 4.2 |  |
|  | OcuCoat | 15 | 1 week | 16.7 | 3.3 | 13.9 | 3.8 |  |
|  | OcuCoat | 15 | 1 month | 16.7 | 3.3 | 13.9 | 2.8 |  |
|  | OcuCoat | 15 | 3 months | 16.7 | 3.3 | 12.4 | 3.0 |  |
|  | Celoftal | 15 | 6 hours | 16.5 | 3.1 | 21.5 | 7.1 |  |
|  | Celoftal | 15 | 24 hours | 16.5 | 3.1 | 19.4 | 3.6 |  |
|  | Celoftal | 15 | 1 week | 16.5 | 3.1 | 12.9 | 2.9 |  |
|  | Celoftal | 15 | 1 month | 16.5 | 3.1 | 13.6 | 3.0 |  |
|  | Celoftal | 15 | 3months | 16.5 | 3.1 | 12.0 | 2.0 |  |
| Hutz (1996) | Methocel | 50 | 6 hours | 16.5 | 3.8 | 19.5 | 6.0 |  |
|  | Methocel | 50 | 5 days | 16.5 | 3.8 | 14.5 | 5.7 |  |
|  | Viscoat | 50 | 6 hours | 16.5 | 3.8 | 18.5 | 5.7 |  |
|  | Viscoat | 50 | 5 days | 16.5 | 3.8 | 15.5 | 5.3 |  |
|  | Healon | 50 | 6 hours | 16.5 | 3.8 | 19.0 | 7.6 |  |
|  | Healon | 50 | 5 days | 16.5 | 3.8 | 15.0 | 4.8 |  |
|  | Healon GV | 50 | 6 hours | 16.5 | 3.8 | 20.0 | 8.4 |  |
|  | Healon GV | 50 | 5 days | 16.5 | 3.8 | 15.0 | 5.5 |  |
| Kim (2004) | Soft shell (Viscoat-Hyal-2000) | 69 | 24 hours | 14.2 | 2.7 | 15.8 | 3.0 | 1.5 (2.4) |
|  | Viscoat | 64 | 24 hours | 14.3 | 2.7 | 15.0 | 3.5 | 1.9 (2.1) |
|  | Hyal-2000 | 64 | 24 hours | 15.2 | 4.5 | 15.3 | 2.5 | 1.8 (1.8) |
|  | Provisc | 55 | 24 hours | 14.2 | 4.5 | 15.5 | 3.6 | 1.4 (1.6) |
| Kocak-Altintas (2007) | BD Visc | 83 | 24 hours | 13.7 | 2.9 | 19.4 | 9.7 |  |
|  | Healon | 83 | 24 hours | 14.3 | 3.2 | 16.8 | 8.9 |  |
| Kohnen (1996) | Healon | 30 | 6 hours | 14.8 | 2.3 | 16.8 | 3.3 | 2.0 (3.6) |
|  | Healon | 30 | 24 hours | 14.8 | 2.3 | 17.7 | 3.7 | 2.9 (4.3) |
|  | Healon | 30 | 2 days | 14.8 | 2.3 | 15.6 | 3 | 0.8 (3.2) |
|  | Healon | 30 | 3 days | 14.8 | 2.3 | 15.5 | 3.4 | 0.7 (3.6) |
|  | Healon | 29 | 1 month | 14.8 | 2.3 | 14.6 | 2.6 | -0.2 (2.6) |
|  | Healon GV | 30 | 6 hours | 14.2 | 2.2 | 16.2 | 5.4 | 2.0 (5.1) |
|  | Healon GV | 30 | 24 hours | 14.2 | 2.2 | 17.5 | 6.8 | 3.3 (6.3) |
|  | Healon GV | 30 | 2 days | 14.2 | 2.2 | 14.3 | 2.3 | 0.1 (2.9) |
|  | Healon GV | 28 | 3 days | 14.2 | 2.2 | 14.3 | 2.5 | 0.1 (3.0) |
|  | Healon GV | 27 | 1 month | 14.2 | 2.2 | 14.1 | 2.8 | -0.3 (2.9) |

| **Author (Year)** | **OVDs** | **N (eyes)** | **Follow-up** | **Mean Pre-op IOP** | **Pre-op IOP (SD)** | **Post-op IOP**  **Mean** | **Post-op IOP (SD)** | **IOPR% [SD_IOPR%_]** |
| --- | --- | --- | --- | --- | --- | --- | --- | --- |
| Lee (2011) | Amvisc plus | 31 | 6 hours | 14.42 | 4.21 | 21.43 | 6.41 |  |
|  | Amvisc plus | 31 | 1 week | 14.42 | 4.21 | 14.42 | 4.21 |  |
|  | Balanced Salt Solution + Amvisc plus | 31 | 6 hours | 13.32 | 5.18 | 17.41 | 5.12 |  |
|  | Balanced Salt Solution + Amvisc plus | 31 | 1 week | 13.32 | 5.18 | 13.32 | 5.18 |  |
| Miyata (2002a) | Opegon | 50 | 24 hours | 11.2 | 3.5 | 10.5 | 4.2 |  |
|  | Opegon | 50 | 1 week | 11.2 | 3.5 | 10.2 | 3.5 |  |
|  | Healon | 28 | 24 hours | 10.9 | 2.8 | 11.2 | 3.0 |  |
|  | Healon | 28 | 1 week | 10.9 | 2.8 | 9.4 | 2.3 |  |
| Neumayer (2008) | Neocrom Cohesive | 29 | 6 hours | 16.8 | 2.5 | 19.8 | 4.1 | 2.2 (3.5) |
|  | Neocrom Cohesive | 29 | 24 hours | 16.8 | 2.5 | 17.6 | 4.1 | 0.9 (4.3) |
|  | Healon | 29 | 6 hours | 16.5 | 2.4 | 18.2 | 3.9 | 1.4 (4.2) |
|  | Healon | 29 | 24 hours | 16.5 | 2.4 | 16.6 | 3.5 | 0.0 (4.2) |
| Ray-Chaudhary (2006) | Ophthalin | 58 | Preop | 15.7 | 2.8 |  |  |  |
|  | Ophthalin | 51 | 4 hours | 15.7 | 2.8 | 23.6 | 9.5 |  |
|  | Ophthalin | 49 | 1 day | 15.7 | 2.8 | 17.6 | 4.7 |  |
|  | Ophthalin | 48 | 1 week | 15.7 | 2.8 | 13.4 | 3.5 |  |
|  | HPMC-Ophtal | 52 | Preop | 15.9 | 3.5 |  |  |  |
|  | HPMC-Ophtal | 50 | 4 hours | 15.9 | 3.5 | 24.0 | 11.4 |  |
|  | HPMC-Ophtal | 50 | 1 day | 15.9 | 3.5 | 21.3 | 8.3 |  |
|  | HPMC-Ophtal | 47 | 1 week | 15.9 | 3.5 | 13.9 | 3.5 |  |
| Rainer (2000)[^8^](#_ENREF_8) | Healon5 | 35 | 6 hours | 14.9 | 2.5 | 20.1 | 6.2 |  |
|  | Healon5 | 35 | 24 hours | 14.9 | 2.5 | 15.3 | 3.9 |  |
|  | Healon5 | 35 | 1 week | 14.9 | 2.5 | 13.5 | 2.3 |  |
|  | Viscoat | 35 | 6 hours | 15.2 | 2.9 | 25.2 | 9.0 |  |
|  | Viscoat | 35 | 24 hours | 15.2 | 2.9 | 15.0 | 3.4 |  |
|  | Viscoat | 35 | 1 week | 15.2 | 2.9 | 13.5 | 2.5 |  |

| **Author (Year)** | **OVDs** | **N (eyes)** | **Follow-up** | **Mean Pre-op IOP** | **Pre-op IOP (SD)** | **Post-op IOP**  **Mean** | **Post-op IOP (SD)** | **IOPR% [SD_IOPR%_]** |
| --- | --- | --- | --- | --- | --- | --- | --- | --- |
| Rainer (2001)[^9^](#_ENREF_9) | OcuCoat | 40 | 6 hours | 14 | 2.8 | 18.5 | 6 | 4.6 (5.1) |
|  | OcuCoat | 40 | 24 hours | 14 | 2.8 | 14 | 3.5 | 0.3 (3.3) |
|  | OcuCoat | 40 | 1 week | 14 | 2.8 | 13.5 | 2.7 | -0.4 (2.0) |
|  | Viscoat | 40 | 6 hours | 14.1 | 2.8 | 22.7 | 8.7 | 8.6 (8.1) |
|  | Viscoat | 40 | 24 hours | 14.1 | 2.8 | 14.7 | 4 | 0.8 (3.0) |
|  | Viscoat | 40 | 1 week | 14.1 | 2.8 | 13.8 | 2.9 | -0.2 (2.3) |
| Rainer (2007) | NaHa 1% | 40 | 30 mins | 14.7 | 2.7 | 15.6 | 5.4 | 0.9 (4.0) |
|  | NaHa 1% | 40 | 1 hour | 14.7 | 2.7 | 16.8 | 5.3 | 2.1 (3.9) |
|  | NaHa 1% | 40 | 2 hours | 14.7 | 2.7 | 18.6 | 6.1 | 3.9 (5.3) |
|  | NaHa 1% | 40 | 3 hours | 14.7 | 2.7 | 18.8 | 7.1 | 4.1 (6.0) |
|  | NaHa 1% | 40 | 4 hours | 14.7 | 2.7 | 18.7 | 6.4 | 4.0 (5.5) |
|  | NaHa 1% | 40 | 6 hours | 14.7 | 2.7 | 19.7 | 7.2 | 5.0 (6.0) |
|  | NaHa 1% | 40 | 8 hours | 14.7 | 2.7 | 20 | 7.5 | 5.3 (6.4) |
|  | NaHa 1% | 40 | 24 hours | 14.7 | 2.7 | 16.5 | 4.3 | 1.9 (3.3) |
|  | NaHa 1% | 40 | 1 week | 14.7 | 2.7 | 15.3 | 3.5 | 0.6 (2.8) |
|  | 2% HPMC | 40 | 30 mins | 14.7 | 3.2 | 18.3 | 7 | 3.7 (6.6) |
|  | 2% HPMC | 40 | 1 hour | 14.7 | 3.2 | 20.5 | 7 | 5.8 (6.2) |
|  | 2% HPMC | 40 | 2 hours | 14.7 | 3.2 | 22.4 | 6.5 | 7.8 (6.1) |
|  | 2% HPMC | 40 | 3 hours | 14.7 | 3.2 | 21.7 | 7.7 | 7.0 (7.4) |
|  | 2% HPMC | 40 | 4 hours | 14.7 | 3.2 | 20.9 | 6.2 | 6.2 (5.8) |
|  | 2% HPMC | 40 | 6 hours | 14.7 | 3.2 | 20.2 | 5.1 | 5.5 (4.9) |
|  | 2% HPMC | 40 | 8 hours | 14.7 | 3.2 | 19.6 | 5.3 | 5.0 (5.1) |
|  | 2% HPMC | 40 | 24 hours | 14.7 | 3.2 | 16.9 | 4.3 | 2.2 (3.9) |
|  | 2% HPMC | 40 | 1 week | 14.7 | 3.2 | 15.5 | 3.4 | 0.9 (3.4) |
| Rainer (2008) | Viscoat | 30 | 1 hour | 15.9 | 3.2 | 25.8 | 7.4 | 9.8 (7.5) |
|  | Viscoat | 30 | 6 hours | 15.9 | 3.2 | 24.7 | 6 | 8.7 (5.3) |
|  | Viscoat | 30 | 24 hours | 15.9 | 3.2 | 17.6 | 6.4 | 1.6 (6.0) |
|  | DuoVisc | 30 | 1 hour | 16.1 | 2.8 | 20.5 | 4.6 | 4.4 (5.0) |
|  | DuoVisc | 30 | 6 hours | 16.1 | 2.8 | 21.1 | 5 | 5.0 (5.2) |
|  | DuoVisc | 30 | 24 hours | 16.1 | 2.8 | 17.2 | 5.7 | 1.1 (6.2) |

| **Author (Year)** | **OVDs** | **N (eyes)** | **Follow-up** | **Mean Pre-op IOP** | **Pre-op IOP (SD)** | **Post-op IOP**  **Mean** | **Post-op IOP (SD)** | **IOPR% [SD_IOPR%_]** |
| --- | --- | --- | --- | --- | --- | --- | --- | --- |
| Schwenn (2000) | Healon5 | 20 | 4 hours | 13.45 | 1.96 | 18.1 | 6.72 |  |
|  | Healon5 | 20 | 8 hours | 13.45 | 1.96 | 16.35 | 5.06 |  |
|  | Healon5 | 20 | 24 hours | 13.45 | 1.96 | 12.25 | 2.97 |  |
|  | Healon5 | 20 | 4 days | 13.45 | 1.96 | 9.55 | 2.19 |  |
|  | Healon5 | 20 | 6 days | 13.45 | 1.96 | 9.84 | 2.19 |  |
|  | Healon5 | 20 | 3 months | 13.45 | 1.96 | 11.06 | 2.41 |  |
|  | Viscoat | 28 | 4 hours | 14.6 | 3.03 | 27.5 | 10.14 |  |
|  | Viscoat | 28 | 8 hours | 14.6 | 3.03 | 23.11 | 8.5 |  |
|  | Viscoat | 28 | 24 hours | 14.6 | 3.03 | 14.54 | 5.48 |  |
|  | Viscoat | 28 | 4 days | 14.6 | 3.03 | 12.25 | 3.52 |  |
|  | Viscoat | 28 | 6 days | 14.6 | 3.03 | 12.21 | 3.83 |  |
|  | Viscoat | 28 | 3 months | 14.6 | 3.03 | 11.42 | 1.96 |  |
| Stankovic (2008) | 2% HPMC | 20 | 6 hours | 14 | 2.8 | 18.6 | 6 |  |
|  | 2% HPMC | 20 | 24 hours | 14.1 | 2.8 | 14 | 3.5 |  |
|  | 2% HPMC | 20 | 3 days | 14.1 | 2.8 | 13.5 | 2.7 |  |
|  | Chondroitin sulphate 4%- NaHa 3% | 20 | 6 hours | 14.1 | 2.8 | 22.7 | 8.7 |  |
|  |  | 20 | 24 hours | 14.1 | 2.8 | 14.7 | 4 |  |
|  |  | 20 | 3 days | 14.1 | 2.8 | 13.8 | 2.9 |  |
| Storr-Paulsen (2007) | Celoftal | 17 | 3 months | 15.1 | 4.6 | - | - | No significant difference |
|  | Vitrax | 16 | 3 months | 16 | 2.7 | - | - |  |
|  | Healon | 19 | 3 months | 16 | 2.4 | - | - |  |
| Strobel (1997) | Healon GV | 30 | 6 hours | 15.4 | 2.8 | 15.6 | 2.7 |  |
|  | Healon | 30 | 6 hours | 15.6 | 2.9 | 16.1 | 2.6 |  |
| Vajpayee (2005) | Viscoat | 19 | 24 hours | 13.3 | 2 | 16 | 4.7 |  |
|  | Viscoat | 19 | 1 week | 13.3 | 2 | 13.8 | 4.6 |  |
|  | Viscoat | 19 | 3 months | 13.3 | 2 | 13 | 3.4 |  |
|  | Healon GV | 19 | 24 hours | 14 | 2.2 | 12.2 | 4.7 |  |
|  | Healon GV | 19 | 1 week | 14 | 2.2 | 12 | 4.5 |  |
|  | Healon GV | 19 | 3 months | 14 | 2.2 | 12.1 | 3.7 |  |
|  | Healon5 | 18 | 24 hours | 13.2 | 3.2 | 12.3 | 4.8 |  |
|  | Healon5 | 18 | 1 week | 13.2 | 3.2 | 12.2 | 4.7 |  |
|  | Healon5 | 18 | 3 months | 13.2 | 3.2 | 12.3 | 3.6 |  |
| Yachimori (2004) | Opegan | 34 | 5 hours | 12.8 | 2.5 | 18.2 | 7.1 |  |
|  | Opegan | 34 | 24 hours | 12.8 | 2.5 | 13.9 | 7.8 |  |
|  | Soft Shell | 35 | 5 hours | 13.0 | 3.1 | 20.7 | 7.0 |  |
|  | Soft Shell | 35 | 24 hours | 13.0 | 3.1 | 15.1 | 3.6 |  |
| OVD = ophthalmic viscoelastic devices; IOP = intraocular pressure; pre-op IOP = pre-operative IOP; post-op IOP = post-operative IOP; IOPR% = percentage reduction in IOP from baseline | | | | | | | | |
